# Evolution of structural neuroimaging biomarkers in a series of adult patients with Niemann-Pick type C under treatment

**DOI:** 10.1186/s13023-017-0579-3

**Published:** 2017-02-02

**Authors:** Marion Masingue, Isaac Adanyeguh, Yann Nadjar, Frédéric Sedel, Damien Galanaud, Fanny Mochel

**Affiliations:** 1 Inserm U 1127, CNRS UMR 7225, Sorbonne Universités, UPMC University Paris 06 UMR S 1127, Institut du Cerveau et de la Moelle épinière, ICM, Paris, France; 2 Department of Neurology, AP-HP, Pitié-Salpêtrière University Hospital, Paris, France; 3 AP-HP, Pitié-Salpêtrière University Hospital, Reference Centre for Lysosomal diseases, Paris, France; 4 MedDay Pharmaceuticals, 96 Boulevard Haussmann, Paris, France; 5 Department of Neuroradiology, AP-HP, Pitié-Salpêtrière University Hospital, Paris, France; 6 Department of Genetics, AP-HP, Pitié-Salpêtrière University Hospital, Paris, France; 7 University Pierre and Marie Curie, Neurometabolic Research Group, Paris, France

**Keywords:** Niemann-Pick type C, Neuroimaging, Biomarkers, Volumetry, Diffusion tensor imaging, Miglustat

## Abstract

**Background:**

Niemann-Pick type C (NPC) disease is a lysosomal storage disorder characterized by a wide clinical spectrum and non-specific conventional magnetic resonance imaging (MRI) signs. As substrate reduction therapy with miglustat is now used in almost all patients, its efficacy and the course of the disease are sometimes difficult to evaluate. Neuroimaging biomarkers could prove useful in this matter. We first performed a retrospective analysis of volumetric and diffusion tensor imaging (DTI) data on 13 adult NPC patients compared to 13 controls of similar age and sex. Eleven NPC patients were then studied using the same neuroimaging modalities over a mean of 5 years. The NPC composite score was used to evaluate disease severity.

**Results:**

NPC patients showed atrophy in basal ganglia – pallidum (*p* = 0.029), caudate nucleus (*p* = 0.022), putamen (*p* = 0.002) and thalamus (*p* < 0.001) – cerebral peduncles (*p* = 0.003) and corpus callosum (*p* = 0.006), compared to controls. NPC patients also displayed decreased fractional anisotropy (FA) in several regions of interest – corona radiata (*p* = 0.015), internal capsule (*p* = 0.007), corpus callosum (*p* = 0.032) and cingulate gyrus (*p* = 0.002) – as well as a broad increase in radial diffusivity (*p* < 0.001), compared to controls. Over time, 3 patients worsened clinically, including 2 patients who interrupted treatment, while 8 patients remained stable. With miglustat, no significant volumetric change was observed but FA improved after 2 years in the corpus callosum and the corona radiata of NPC patients (*n* = 4; *p* = 0.029) – although that was no longer observed at further time points.

**Conclusion:**

This is the first study conducted on a series of adult NPC patients using two neuroimaging modalities and followed under treatment. It confirmed that NPC patients displayed cerebral atrophy in several regions of interest compared to controls. Furthermore, miglustat showed an early effect on diffusion metrics in treated patients. DTI can detect brain microstructure alterations caused by neurometabolic dysfunction. Its potential as a biomarker in NPC shall be further evaluated in upcoming therapeutic trials.

**Electronic supplementary material:**

The online version of this article (doi:10.1186/s13023-017-0579-3) contains supplementary material, which is available to authorized users.

## Background

Neurometabolic disorders encompass a very wide spectrum of diseases, of heterogeneous clinical and radiological presentations. While their diagnosis remains difficult, treatments are emerging, emphasizing the need of non-invasive techniques to evaluate their efficacy. Studying neurometabolic diseases also contributes to the better understanding of neurodegenerative disorders related to metabolic dysfunction such as Parkinson disease [1] or Huntington disease [2]. Niemann-Pick type C disease (NPC) is a genetic lysosomal lipid storage disorder of autosomal recessive inheritance. It results from the mutation of either *NPC1* (95%) or *NPC2* (5%). The resulting impairment of the processing and utilization of endocytosed cholesterol leads to the accumulation of free cholesterol and glycosphingolipids in many tissues, including the brain. Its incidence is difficult to evaluate precisely, and lies around 1 in 120,000 births, but is probably underestimated, particularly in the adult population [3].

Diverse clinical presentations exist, ranging from a very severe perinatal form to a neurodegenerative adult-onset form. Neurological symptoms include mental retardation, cerebellar ataxia with dysarthria and dysphagia, dementia and vertical supranuclear gaze palsy (highly evocative). Psychiatric disorders, epilepsy and dystonia may also occur. In adults, isolated ataxic and/or psychiatric forms have been described, underlining the complexity of the diagnosis. Visceral involvement (liver, spleen, sometimes lungs) is also rather frequent [3, 4]. The diagnosis of NPC was historically made using fillipin staining but is now currently based on elevated plasma oxysterols (cholesterol oxidation products) [5], and confirmed by the molecular analysis of *NPC1* and *NPC2* genes. A substrate reduction therapy exists for NPC patients using miglustat, an inhibitor of glucosylceramide synthase, hence preventing glycosphingolipids accumulation. Miglustat has shown a partial clinical efficiency [6, 7].

Conventional cerebral magnetic resonance imaging (MRI) can be normal in NPC patients or shows unspecific cortical or cerebellar atrophy [8, 9]. White matter abnormalities may be present on T2-weighted images, especially in the severe infantile form [3, 10]. Cerebral volumetry [8, 9, 11] and diffusion tensor imaging (DTI) studies have been performed in NPC [9, 11, 12], but rarely in combination [9, 12] and mainly in children, and few neuroimaging studies have assessed the benefit of treatment in NPC [8, 12, 13]. Accordingly, we performed a retrospective study using both volumetry and DTI on a large cohort of adult patients with NPC at baseline, compared to controls, and under treatment.

## Methods

### Patients and controls

Initial and follow-up information was obtained retrospectively. All participants provided written informed consent for study procedures and data reporting. NPC patients were followed between 2006 and 2016 in the Reference Centre for Lysosomal diseases, and enrolled sequentially. The *NPC1* and *NPC2* genes were sequenced as described [14]. Patients’ functional disability was evaluated at each clinical visit using a disease-specific disability scale for NPC, previously validated [15]. Briefly, 6 key domains were assessed – ambulation, manipulation, language, swallowing, ocular movements and epilepsy. A composite overall score was calculated as the sum of all 6 individual domains and scores could range from 0 (best) to 24 (worst). The frequency of clinical and radiological follow-up was the referring neurologist’s choice at the time, and ranged from 6 to 12 months, in line with recommendations on clinical management of NPC [16]. Clinical score and MRI data were retrieved at baseline before any treatment by miglustat (Y0), and after 1 (Y1), 1.5 (Y1.5), 2 (Y2), 3 (Y3), 4 (Y4), 4.5 (Y4.5), 5 (Y5) and 9 (Y9) years of treatment. If treatment by miglustat was interrupted, clinical and MRI data were retrieved at 1.5 and 4.5 years after treatment initiation. Controls were healthy subjects who were selected in order to be of similar age to patients. They were part of a control cohort built up for a previous DTI study, and were chosen in order to have a similar mean age, sex ratio and ethnicity than our patients [17].

### Brain imaging

MRI acquisitions were performed on 1.5 and 3 T MR units (General Electric, I, USA) using a standard protocol applied for the exploration and follow-up of neurometabolic patients at our institution. A standard 3D T1-weighted image (TR = 9.5 ms, TE = 3 ms, matrix = 256 × 256, field of view (FOV) = 256 × 256 (*n* = 77), 250 × 250 or 512 × 512 (*n* = 34), 240 × 240 or 220 × 220) was acquired for localization of brain regions and volumetric analysis. DTI was performed in some patients (*b* value = 1000 s/mm^2^, 12 directions (6 directions in 2 patients), matrix = 256 × 256, FOV either 240 × 240, 280 × 280 or 380 × 380, TR = 12000 ms, TE = 80 ms) to evaluate the integrity of white matter microstructure. Volumetry and DTI analysis were run using the FMRIB Software Library (FSL) (https://www.fmrib.ox.ac.uk/fsl).

The voxel-based-morphometry (VBM) tool of FSL was used on the 3D T1 images for brain extraction and segmentation. The segmented images were then non-linearly registered to a study-specific grey matter template, smoothed and a statistic map was created using a threshold-free cluster enhancement (TFCE) approach. Region of interest (ROI) analysis was also performed, and segmentation of cortical and subcortical structures was automated with FreeSurfer 5.3 (https://surfer.nmr.mgh.harvard.edu). Brain volumes were normalized to the total intracranial volume of each subject to eliminate the bias from differences in skull sizes.

For DTI analyses, quality control of DTI images was performed by visual inspection for spikes and other artifacts. Eddy correction was performed to correct for the effects of head movement and eddy current induced geometric distortions. DTI provides information on microstructure integrity using metrics such as fractional anisotropy (FA) and radial diffusivity (RD). FA measures the global diffusion where lower values correspond to reduced diffusion and thus reduced fiber bundles. RD measures diffusion across the fibers and increased values usually indicate myelin injury. The diffusion tensor model was fitted to generate FA maps. Thus, we extracted FA and other diffusion metrics such as RD and axial diffusivity from different ROI (based on the JHU-atlas). We also performed statistical analysis using the voxel-wise manner with the tract based spatial statistics (TBSS) tool of FSL. First, the FA-maps were non-linearly transformed to the MNI space, and an average FA image corresponding to the mean FA was created. A threshold skeletonized mean FA image was created onto which all subjects’ FA was projected before cross-subject voxel-wise statistics were performed using FSL’s *randomise*. Multiple comparison correction was performed with a threshold of *p* < 0.05. Radial diffusivity data were studied in the same manner.

### Quality control

All MRI were acquired during routine clinical follow-up. This resulted at times in a loss of quality, precision and homogeneity compared to research MRI, as illustrated by the difference in FOV and matrix across images. All images were visually and manually checked and not included if quality was inappropriate: (i) only axial or non-3D images making segmentation not possible or unreliable (*n* = 2); (ii) images with severe motion artifacts that made them too poor to evaluate (*n* = 1 for T1 analysis and *n* = 7 for DTI).

### Statistical analyses

Means were compared with Mann and Whitney test, correlations were studied with Spearman coefficient. For multiple tests, the Holm-Bonferroni method of correction was applied. To compare MRI data under treatment at different times and with untreated patients, we used homogenous groups of patients who had several MRIs-for instance, patients with only one baseline MRI were compared to controls but excluded from the “baseline” group for comparisons with treated patients over time.

## Results

At baseline, volumetric data were available for 13 NPC patients while DTI metrics were available for 8 NPC patients. Their clinical and demographic characteristics are shown on Table 1. Eleven NPC patients were then followed over a mean of 5 years (1–9 years) with MRI available at different time-points, as one patient had just been diagnosed and another patient was lost to follow up. These 11 patients were treated with miglustat, but two interrupted their treatment after 6 months, the first patient because of a severe weight loss and the second patient due to major digestive and psychiatric side effects. Follow-up time extended up to 9 years. The 2 patients who interrupted treatment had baseline MRI, and radiological follow-up at 1.5 and 4.5 years. Among all patients with MRI available for follow-up, 3 worsened clinically: one with sustained miglustat therapy and the 2 patients who interrupted treatment. All of the others had a global stabilization-i.e., loss or gain in overall score ≤1 (Table 2).

**Table 1:** Characteristics of NPC patients at baseline

	Volumetry (*n* = 13)	DTI (*n* = 8)
Age (years) (±SD, min-max)	35 (±14, 20–65)	35 (±14, 20–65)
Age at onset (years)	18 (±14, 5–56)	16 (±10, 5–30)
Disease evolution prior MRI (years)	17 (±12, 2–35)	19 (±14, 2–35)
NPC composite score	9 (±3, 3–15)	9 (±3, 3–15)

*SD*: standard deviation; *M*: male; *F*: female. Disease evolution prior to MRI represents disease duration (i.e., time from first symptom) before baseline MRI

**Table 2:** Clinical evolution of NPC patients using the NPC composite score [12]

Patient	1	2	3	4	5	6	7	8	9	10	11
Baseline NPC score	10	10	9	7	3	5	10	11	10	10	15
Last NPC score	10	13	8	10	8	5	10	12	10	10	15
Time range (year)	5	9	4	4	4.5	1	9	2	5	2	9
Sustained treatment	+	+	+	-	-	+	+	+	+	+	+

Eleven patients had a clinical follow-up ranging from 1 to 9 years. Clinical stabilization was defined as a variation in the total score ≤ 1. Patients 2, 4 and 5 worsened, but only patient 2 had not interrupted treatment with miglustat

**Fig. 1:**
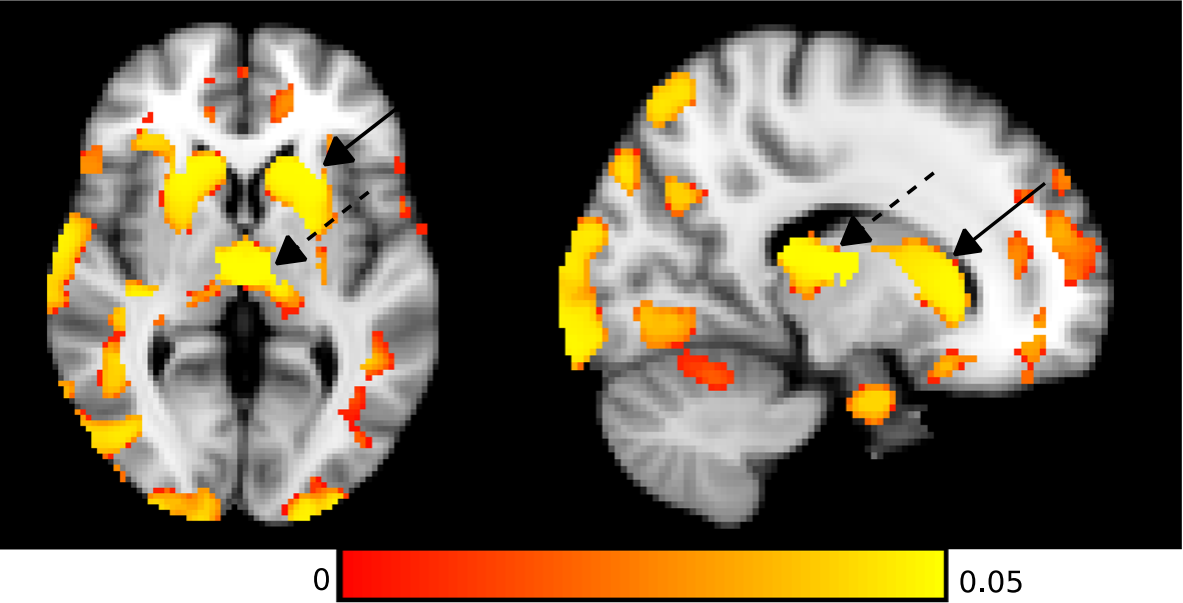
Atrophy of the basal ganglia from NPC patients compared to healthy controls. Statistical map using FSL-VBM showed voxels with significant volume changes in the caudate nucleus (solid arrow) and the thalamus (dotted arrow)

**Fig. 2:**
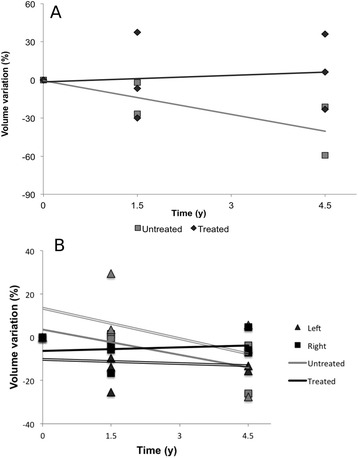
**a** Volume changes over time in the corpus callosum, according to treatment. Atrophy tended to be faster in the 2 untreated patients compared to the 8 patients treated with miglustat; **b** Volume changes over time in the caudate nucleus, according to treatment. The 2 untreated patients tended to have a faster basal ganglia atrophy compared to the 8 patients treated with miglustat

For volumetric analyses at baseline, the control group (*n* = 13) had a mean age of 35 (±12) years and a sex-ratio comparable to patients (M/F: 7/6). Eight NPC patients had radiological follow-up at 1 (*n* = 6), 1.5 (*n* = 3), 2 (*n* = 6), 3 (*n* = 3), 4 (*n* = 4), 4.5 (*n* = 3), 5 (*n* = 4) or 9 (*n* = 3) years. Analyses with VBM and ROI methods both showed global atrophy and decreased volume in the basal ganglia of NPC patients compared to controls (Fig. 1, Additional file 1: Table S1). The mean volume (% of total intracranial volume ± SD [min-max]) of superior cerebral peduncles was smaller in the patients group compared to controls – 0.01% ±0.002 [0.01–0.02] vs. 0.02% ± 0.002 [0.01–0.02], *p* = 0.003 – as well as the anterior corpus callosum – 0.04% ± 0.01 [0.02–0.07] vs. 0.06% ±0.01 [0.04–0.09], *p* = 0.006. When looking at the evolution of the volume of regions of interest in patients treated with miglustat, a significant atrophy was found over time in the thalamus, pallidum, and amygdala (data not shown). There was a tendency to slower atrophy in caudate nucleus, putamen and corpus callosum in treated patients (*n* = 3) compared to patients who withdrew treatments (*n* = 2) but that was not significant (Fig. 2a and b). We found no significant correlations between volume changes and clinical scores. There was no significant difference either in volume changes between patients who worsened clinically and patients who stabilized.

**Fig. 3:**
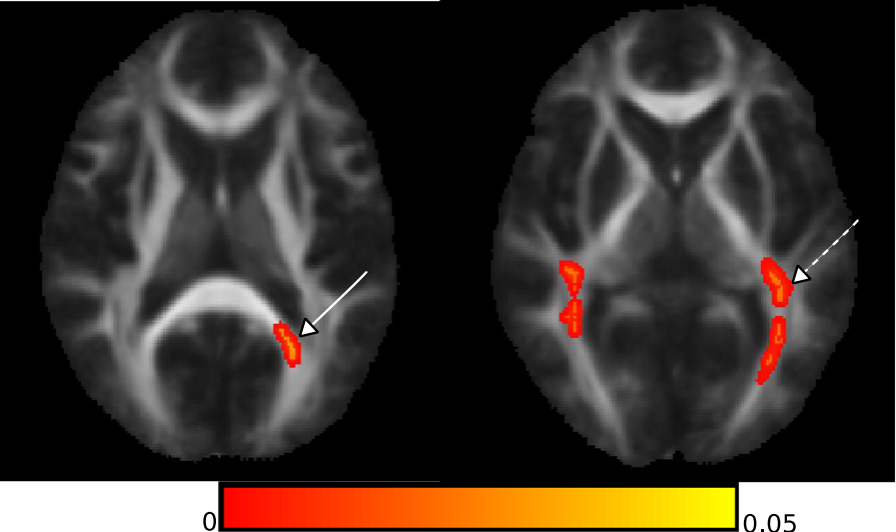
Reduced FA in NPC compared to controls. Statistical map using FSL-TBSS showed voxels with significantly decreased FA in the posterior corona radiata (dotted arrow) and the forceps major of the corpus callosum (solid arrow)

**Fig. 4:**
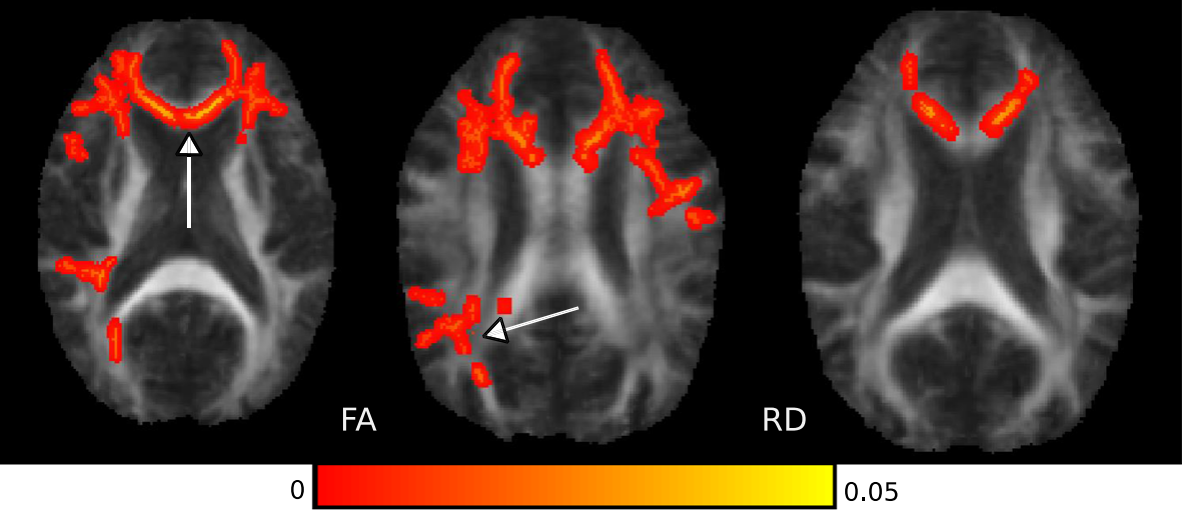
FA increase and RD decrease after 2 years of treatment with miglustat. Statistical map using FSL-TBSS showed voxels with significant differences in FA and RD, at baseline and after 2 years of treatment, in the corpus callosum (arrow in the left FA image and RD) and the superior region of corona radiata (arrow in the right FA image)

**Fig. 5:**
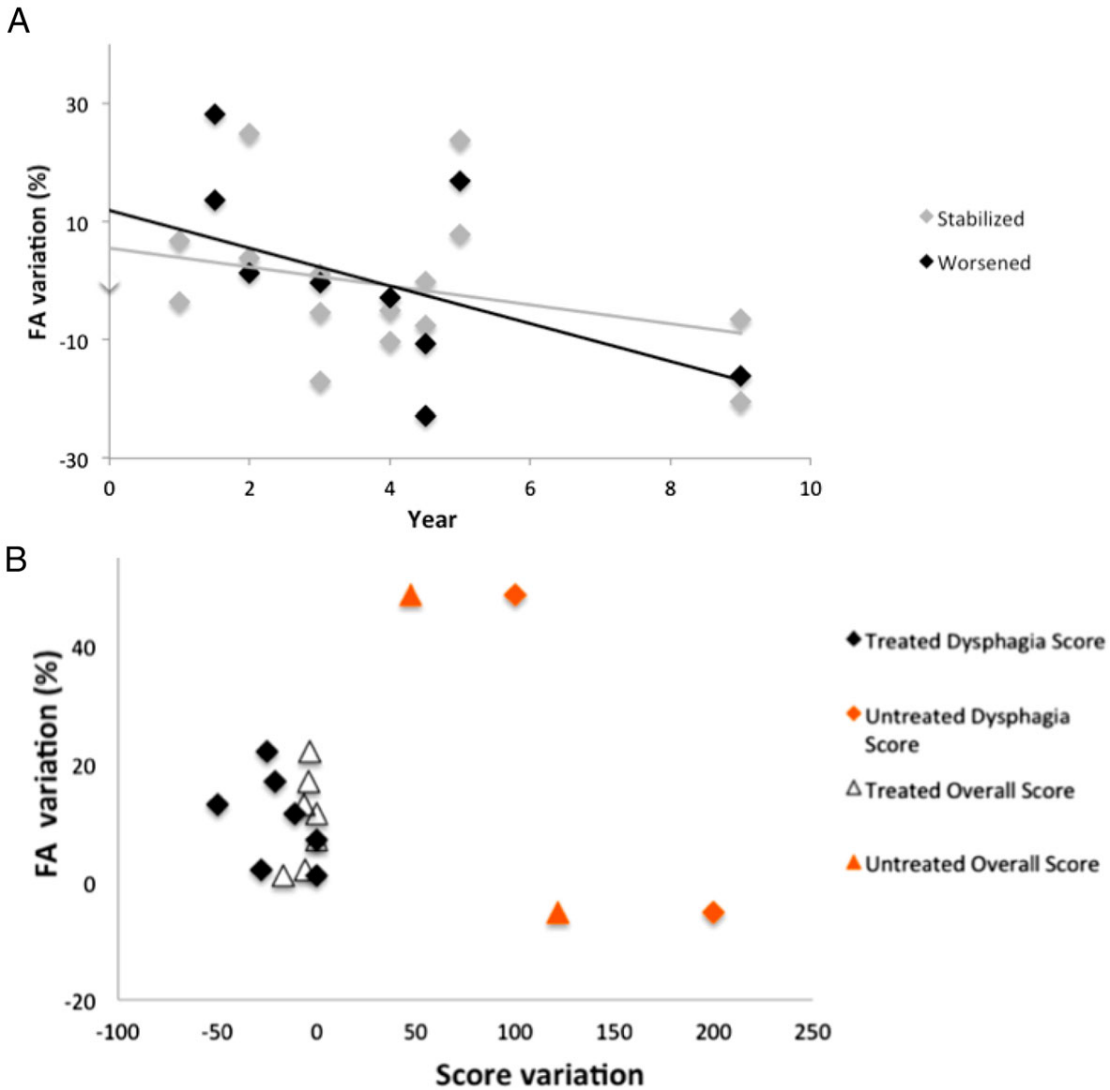
**a** FA variation in NPC patients who deteriorated over time versus stabilized patients. The 8 stable patients tended to have a slower FA decrease than the 3 patients who deteriorated. **b** FA and clinical variations in NPC patients according to treatment. The 2 untreated patients tended to present with larger FA variations, along with clinical aggravation, compared to the 8 patients treated with miglustat who featured relatively minimal FA and clinical score variations

For DTI analyses, the control group (*n* = 8) had a mean age of 34 years (±14), and a sex-ratio comparable to patients (M/F: 5/3). Four NPC patients had radiological follow-up at 1, 2, 3, 4 (*n* = 4), 4.5 (*n* = 2), 5 (*n* = 4) and 9 (*n* = 3) years. At baseline, TBSS analyses found significantly decreased FA in the posterior corona radiata and left forceps major (Fig. 3). ROI analyses found a significantly decreased FA in the corpus callosum, anterior corona radiata and internal capsule, posterior thalamic radiations, cerebellar peduncles, cingulate gyrus, and longitudinal and fronto-occipital fasciculii (Table 3). RD was globally increased in NPC patients compared to patients (0.00062 ± 0.00003 [0.00064–0.00065] vs. 0.00075 ± 0.00007 [0.00070–0.00081, *p* < 0.001). After 2 years of treatment with miglustat, TBSS analyses showed significantly increased FA in the corpus callosum, forceps minor and superior region of corona radiata, as well as significantly decreased RD in the corpus callosum (Fig. 4). ROI analyses also showed increased FA after 2 years of treatment in the right hippocampal cingulum (0.321 ± 0.170 vs 0.308 ± 0.546, *p* = 0.029) and in the anterior corona radiata (0.431 ± 0.178 vs 0.349 ± 0.180, *p* = 0.029). However, such difference was no longer observed at further time points (years 3, 4, 5 and 9) compared to baseline MRI. FA in the corona radiata tended to worsen more rapidly in non-responding or untreated patients (Fig. 5a). Moreover, the overall score and the dysphagia score seemed more likely to stabilize if the change in FA was minor (Fig. 5b).

**Table 3:** Changes in FA in NPC patients compared to controls

ROI		NPC (m ± SD [min-max])	Controls (m ± SD [min-max])	*p*
Global mean		0.436 ± 0.023 [0.391–0.468]	0.469 ± 0.023 [0.423_0.502]	*0.028*
Anterior corona radiata	L	0.365 ± 0.048 [0.299–0.446]	0.405 ± 0.030 [0.365–0.444]	*0.083*
	R	0.358 ± 0.035 [0.316–0.421]	0.409 ± 0.031 [0.438–0.531]	*0.015*
Anterior limb of internal capsule	L	0.449 ± 0.029 [0.409–0.507]	0.484 ± 0.029 [0.456–0.543]	*0.028*
	R	0.450 ± 0.025 [0.415–0.496]	0.500 ± 0.029 [0.456–0.543]	*0.007*
Cingulate gyrus	L	0.386 ± 0.027 [0.342–0.424]	0.431 ± 0.031 [0.392–0.467]	*0.002*
	R	0.359 ± 0.025 [0.321–0.389]	0.409 ± 0.024 [0.373–0.444]	*0.015*
Body corpus callosum		0.473 ± 0.044 [0.408–0.533]	0.531 ± 0.054 [0.448–0.608]	*0.032*

ROI analysis found significantly decreased FA in NPC patients in the corpus callosum, the corona radiata, the internal capsule and the cingulate gyrus in particular. *m* : mean; *SD* : standard deviation; *min* : minimum; *max* : maximum, *L* : left; *R*: right

## Discussion

The novelty of our work lies in the following points: (i) this study is the largest performed in adult NPC patients using two different neuroimaging modalities; (ii) the whole brain was analyzed and not only regions of interest; (iii) most patients had follow-up scans under treatment. As suggested by visual inspection from brain MRI of NPC patients [4, 8–10], our volumetric analyses showed that NPC patients display cerebral atrophy compared to healthy controls, especially the corpus callosum and the cerebral peduncles. Treatment with miglustat failed to prevent further atrophy of the basal ganglia over time. However, it is unclear how volume changes reflect disease severity and/or therapeutic efficacy in NPC since no correlation between clinical and MRI metrics were found in our cohort. Likewise, changes in brain volumes and clinical expression of neurological conditions are not always related as shown in neurodegenerative disorders such as Alzheimer disease [18]. Thus, it remains to be established whether brain volumetry can generate clinically relevant biomarkers in NPC, especially in longitudinal research studies. On the other hand, DTI analyses showed decreased FA in NPC patients compared to controls, especially in the corpus callosum, as previously shown [12, 19], but also the internal capsule, the corona radiata and the cingulate gyrus-with a possible correlation with clinical manifestations for the two latter. The greater changes observed in RD suggest a preferential alteration of the myelin sheath in NPC patients. Overall, the DTI changes identified in NPC patients in key white matter regions may directly reflect cerebral metabolic alterations. These data are coherent with recent work in NPC dedicated to white matter analysis [20]. Of note, treatment with miglustat led to an early, although transient, improvement of DTI metrics.

This study has some limitations. First, due to their acquisition in a clinical setting, some data did not pass quality control requirements and were discarded. Second, combining data from different field strengths can bias the outcome of semi-automated methods such as VBM analyses. Still, data acquisition in clinical routine can be implemented in a multicentric setting with a more systematic radiological follow-up. Third, the sample size of the study is relatively small, a problem inherent to any rare neurometabolic disease, although greater than previously reported in adults with NPC [8, 9, 12]. Last, it can be difficult to establish the relevance of neuroimaging parameters in NPC as (i) there is a lack of natural history data prior to miglustat, (ii) a vast majority of patients are now treated with miglustat, preventing us from assembling an untreated control group and, (iii) the clinical impact of miglustat is relatively weak [6, 7], so that a strong biomarker effect is less likely.

## Conclusion

Overall this work suggests that DTI can provide relevant biomarkers of disease progression in NPC, including in a clinical setting, but should be confirmed in prospective studies, especially if drugs with a greater effect on disease progression become available.

## Additional file

Additional file 1: Table S1. Changes in volumetry in NPC patients compared to controls. ROI analysis found significantly decreased volumes in NPC patients in the corpus callosum, the basal ganglia and the superior cerebral peduncle in particular. m : mean; SD : standard deviation; min : minimum; max : maximum, L : left; R: right. (DOCX 82 kb)

## Abbreviations

DTI: Diffusion tensor imaging
FA: Fractional anisotropy
NPC: Niemann-Pick type C
RD: Radial diffusivity
