# Supplementary material for: Evolution of structural neuroimaging biomarkers in a series of adult patients with Niemann-Pick type C under treatment
Source: Orphanet J Rare Dis. 2017 Feb 2;12:22. doi: 10.1186/s13023-017-0579-3 (PMC5289046; doi:10.1186/s13023-017-0579-3)
Supplement: Additional file 1: Table S1. — Changes in volumetry in NPC patients compared to controls. ROI analysis found significantly decreased volumes in NPC patients in the corpus callosum, the basal ganglia and the superior cerebral peduncle in particular. m : mean; SD : standard deviation; min : minimum; max : maximum, L : left; R: right. (DOCX 82 kb) [file 13023_2017_579_MOESM1_ESM.docx]

**Table S1: Changes in volumetry in NPC patients compared to controls.** ROI analysis found significantly decreased volumes in NPC patients in the corpus callosum, the basal ganglia and the superior cerebral peduncle in particular. m : mean; SD : standard deviation; min : minimum; max : maximum, L : left; R: right.

| **ROI** |  | **NPC**  (m% ± SD [min-max]) | **Controls**  (m% ± SD [min-max]) | ***p*** |
| --- | --- | --- | --- | --- |
| 3^rd^ Ventricle |  | 0.1±0.04 [0.04-0.2] | 0.07±0.01 [0.04-0.1] | *0.003* |
| 4^th^ Ventricle |  | 0.2±0.04 [0.1-0.2] | 0.1±0.05 [0.09-0.3] | *0.019* |
| Putamen | L | 0.3±0.1 [0.1-0.6] | 0.4±0.04 [0.3-0.4] | *0.002* |
|  | R | 0.3±0.1 [0.1-0.5] | 0.4±0.04 [0.3-0.4] | *0.009* |
| Caudate | L | 0.2±0.05 [0.2-0.3] | 0.2±0.02 [0.2-0.3] | *0.022* |
|  | R | 0.2±0.07 [0.1-0.4] | 0.3±0.02 [0.2-0.3] | *0.022* |
| Thalamus | L | 0.4±0.08 [0.3-0.6] | 0.5±0.03 [0.4-0.6] | *<0.001* |
|  | R | 0.4±0.05 [0.3-0.4] | 0.5±0.02 [0.46-0.53] | *<0.001* |
| Anterior Corpus callosum |  | 0.04 ± 0.01 [0.02-0.07] | 0.06±0.01 [0.04-0.09] | *0.006* |
| Superior Cerebral Peduncle |  | 0.01 ±0.002 [0.01-0.02] | 0.02±0.002 [0.01-0.02] | *0.029* |
